# Ultrafast dynamical Lifshitz transition

**DOI:** 10.1126/sciadv.abd9275

**Published:** 2021-04-21

**Authors:** Samuel Beaulieu, Shuo Dong, Nicolas Tancogne-Dejean, Maciej Dendzik, Tommaso Pincelli, Julian Maklar, R. Patrick Xian, Michael A. Sentef, Martin Wolf, Angel Rubio, Laurenz Rettig, Ralph Ernstorfer

**Affiliations:** 1 Fritz Haber Institute of the Max Planck Society, Faradayweg 4-6, Berlin 14195, Germany.; 2 Max Planck Institute for the Structure and Dynamics of Matter, Luruper Chaussee 149, Hamburg 22761, Germany.; 3 Department of Applied Physics, KTH Royal Institute of Technology, Hannes Alfvéns väg 12, 114 19 Stockholm, Sweden.; 4 Center for Computational Quantum Physics (CCQ), Flatiron Institute, 162 Fifth Avenue, New York, NY 10010, USA.

## Abstract

Fermi surface is at the heart of our understanding of metals and strongly correlated many-body systems. An abrupt change in the Fermi surface topology, also called Lifshitz transition, can lead to the emergence of fascinating phenomena like colossal magnetoresistance and superconductivity. While Lifshitz transitions have been demonstrated for a broad range of materials by equilibrium tuning of macroscopic parameters such as strain, doping, pressure, and temperature, a nonequilibrium dynamical route toward ultrafast modification of the Fermi surface topology has not been experimentally demonstrated. Combining time-resolved multidimensional photoemission spectroscopy with state-of-the-art TDDFT+*U* simulations, we introduce a scheme for driving an ultrafast Lifshitz transition in the correlated type-II Weyl semimetal *T*_d_-MoTe_2_. We demonstrate that this nonequilibrium topological electronic transition finds its microscopic origin in the dynamical modification of the effective electronic correlations. These results shed light on a previously unexplored ultrafast scheme for controlling the Fermi surface topology in correlated quantum materials.

## Teaser

We demonstate a non-equilibrium route for ultrafast modification of Fermi surface topology in quantum materials.

## INTRODUCTION

The free quantum electron gas approximation within the Drude-Sommerfeld model of metallic solids leads to purely parabolic band dispersion and spherical Fermi surfaces. However, electron-lattice and electron-electron interactions break the quadratic energy-momentum relationship, leading to complex electronic band dispersion and Fermi surface shape and topology. The Fermi surface, which separates unoccupied from occupied electronic states, is of paramount importance to understand a broad range of phenomena in metallic and semimetallic systems. To cite the pioneering work of Kaganov and Lifshitz, “the Fermi surface is the stage on which the drama of the life of the electron is played out” (*1*).

Equilibrium tuning of static macroscopic parameters such as temperature (*2*–*4*), pressure (*5*, *6*), strain (*7*, *8*), external magnetic fields (*9*), or doping (*10*, *11*) have all been demonstrated to be capable of modifying electronic band structures, which is, in some prominent cases, accompanied by an abrupt change in the Fermi surface topology, a phenomenon known as Lifshitz transition (*12*). A Lifshitz transition is often concurrent with strong modifications of material properties since low-energy excitations, relevant to many electronic, magnetic, and optical properties, occur nearby the Fermi surface boundary. For example, this electronic topological transition often leads to a sudden change of the transport properties (*13*, *14*). Moreover, it was demonstrated that Lifshitz transitions coincide with the onset of superconductivity in electron-doped iron arsenic superconductors (*10*). While inducing Lifshitz transitions using equilibrium tuning methods is now well established and understood, a protocol for ultrafast Fermi surface topology engineering would be highly desirable, since it could allow controlling material properties on unprecedented time scales. However, until now, this goal remains elusive.

Here, we experimentally and theoretically demonstrate a route to induce ultrafast Lifshitz transitions in correlated materials based on transient band structure modifications upon the interaction with ultrashort laser pulses. We apply this scheme to the topological type-II Weyl semimetal *T*_d_-MoTe_2_, a material that is known to be in the vicinity of a Coulomb interaction–induced Lifshitz transition (*15*). Experimentally, we directly map the dynamical evolution of its band structure and its Fermi surface on ultrafast time scales using state-of-the-art time-resolved multidimensional photoemission spectroscopy (*16*). Theoretically, we investigate the microscopic origin of the nonequilibrium Lifshitz transition using extensive time-dependent self-consistent Hubbard *U* calculations (TDDFT+*U*) (*17*–*19*). In striking contrast to statically induced Lifshitz transitions, we here show a disruptive out-of-equilibrium strategy to control Fermi surface topology on unprecedented time scales.

**Fig. 1:**
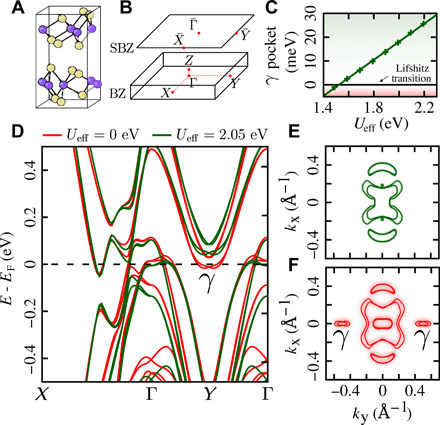
Coulomb-interaction–induced Lifshitz transition in topological Weyl semimetal *T*_d_-MoTe_2_. (**A**) A schematic of the crystal structure of the low-temperature orthorhombic *T*_d_-phase of MoTe_2_ and (**B**) its associated Brillouin zone (BZ) and surface Brillouin zone (SBZ). (**C**) The equilibrium phase diagram revealing the effect of the Hubbard *U*_eff_ = *U* − *J* (*J* being the Hund’s exchange) on the position of the γ pocket relative to the Fermi energy. The dots are data points obtained from DFT+*U* simulations, whereas the thick line is a linear fit to the data, serving as a guide to the eye. (**D**) The electronic band structure of *T*_d_-MoTe_2_ for the equilibrium self-consistently calculated value of *U*_eff_ = 2.05 eV (in green) and for the reduced value of *U*_eff_ = 0 eV (below the adiabatic Lifshitz transition) and their respective Fermi surface cuts taken at *k*_z_ = 0 (**E** and **F**). In (F), one can clearly see the hallmark of the Coulomb-induced Lifshitz transition—the appearance of γ electron pockets at the Fermi surface.

*T*_d_-MoTe_2_ belongs to the transition metal dichalcogenide (TMDC) family. TMDCs have recently attracted tremendous interest mostly because of the unique combination of strong spin-orbit splitting, locally broken inversion symmetry, and enhanced electronic and mechanical properties, making them very interesting for fundamental studies and for applications in electronics, spintronics, optoelectronics, and twistronics. In particular, when cooled below ∼250 K, MoTe_2_ is known to undergo a structural phase transition from the monoclinic and topologically trivial semimetallic (*1T*′) phase to the orthorhombic type-II Weyl semimetallic (*T*_d_-) phase (Fig. 1, A and B), characterized by tilted Weyl cones originating from a protected crossing between the valence and conduction bands in reciprocal space (*20*). Weyl points act as a topological charge, i.e., either a source or a sink of Berry curvature, leading to many fascinating physical phenomena, including the emergence of Fermi arcs (*21*, *22*) and various transport anomalies (*23*). Elucidating the detailed electronic structure of this prominent two-dimensional (2D) Weyl semimetal has been the subject of a lot of experimental and theoretical efforts. Recently, combined static soft x-ray angle-resolved photoemission spectroscopy (ARPES) and density functional theory (DFT) +*U* studies reported that the inclusion of corrected on-site Coulomb interaction (Hubbard *U*) is essential to reproduce the experimentally measured electronic band structure and Fermi surface of *T*_d_-MoTe_2_ (*15*, *24*).

## RESULTS AND DISCUSSION

Figure 1D shows the calculated band structures for different values of the effective Hubbard *U*_eff_: in green for the self-consistent value (*U*_eff_ = 2.05 eV) obtained using our first-principle method (*17*) and in red for a reduced *U*_eff_ = 0 eV. While the modification of the effective on-site Coulomb interaction leaves the band dispersions along Γ-X mostly unchanged, it leads to a substantial energy shift of two slightly spin-orbit split electron pockets (separated by 36.5 meV) located around the *Y* high-symmetry points. Figure 1C shows the energy position of the lower-lying γ pocket as a function of the effective Hubbard *U*_eff_. Around *U*_eff_ ∼ 1.5 eV, the γ pocket is crossing the Fermi level, leading to a Lifshitz transition, as evidenced by the Fermi surface cuts depicted in (Fig. 1 (E and F)), in good agreement with the predictions of Xu *et al.* (*15*). Because *T*_d_-MoTe_2_ is in the vicinity of a Coulomb-induced Lifshitz transition, it is an interesting candidate for investigating the possibility to control the Fermi surface topology on ultrafast time scales.

**Fig. 2:**
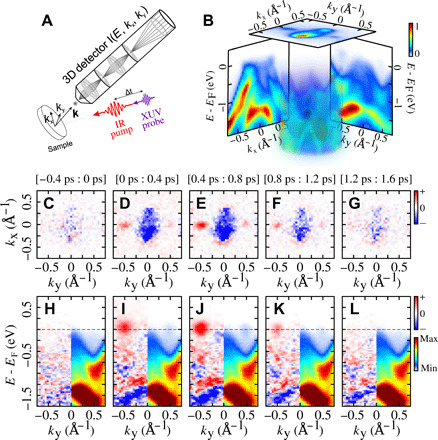
Ultrafast dynamical Lifshitz transition probed by time-resolved multidimensional photoemission spectroscopy. (**A**) A schematic of the experimental setup featuring infrared (IR) pump/extreme ultraviolet (XUV) probe pulses and a time-of-flight momentum microscope detector allowing for parallel measurement of the band structure of the crystalline solid, as a function of pump-probe time delay (*E*_B_, *k*_x_, *k*_y_, Δ*t*). (**B**) An example of the experimental 3D volumetric photoemission data, as well as cuts along different high-symmetry directions and cut at the Fermi energy, integrated for all positive time delays. (**C** to **G**) Differential (unpumped signal subtracted) 2D Fermi surfaces (*k*_x_, *k*_y_) as a function of time delay between the IR pump and the XUV probe (integrated more than 400 fs intervals). (**H** to **L**) Corresponding raw (right, *k*_y_ > 0) and differential (left, *k*_y_ < 0) energy-resolved cuts along *Y*-Γ-*Y* (*k*_x_ = 0).

We used time-resolved multidimensional photoemission spectroscopy to directly probe the nonequilibrium electronic structure of *T*_d_-MoTe_2_. Our experimental setup includes a monochromatized high-order harmonic generation (HHG)–based extreme ultraviolet (XUV) source at 500-kHz repetition rate and centered around 21.7 eV [bandwidth of 110-meV full width at half maximum (FWHM) and pulse duration of ∼20-fs FWHM] (*25*), spanning the full extent of the surface Brillouin zone in parallel momentum. Both infrared (IR) pump (1030 nm, 140-fs FWHM, 6.7 × 10^9^ W/cm^2^) and XUV probe are p polarized and focused onto the *T*_d_-MoTe_2_ sample, handled, and cooled to 30 K by a six-axis cryogenic manipulator. The photoemitted electrons are collected by a time-of-flight momentum microscope (*26*), a multidimensional detection scheme to obtain the 4D (*E*_B_, *k*_x_, *k*_y_, Δ*t*) nonequilibrium electronic structure (Fig. 2, A and B) (*16*). We call this technique time-resolved multidimensional photoemission spectroscopy since we directly measure 4D photoemission intensity I(*E*_B_, *k*_x_, *k*_y_, Δ*t*), instead of the more standard 3D photoemission intensity I(*E*_B_, *k*_‖_, Δ*t*), when using hemispherical analyzer. More information about the experimental setup can be found in Materials and Methods and in (*27*).

We tracked the evolution of the Fermi surface as a function of pump-probe delay (Fig. 2, C to G). After the interaction with the femtosecond IR pump pulse, the appearance of the γ pockets is visible (Fig. 2D) on the Fermi surface. This is the hallmark of the dynamical Lifshitz transition. At longer delays, the signal in the γ pockets at the Fermi energy continues to increase (Fig. 2E), followed by gradual decays (Fig. 2F), and eventually vanish after <1 ps (Fig. 2G), indicating the recovery of the equilibrium Fermi surface topology.

What is the origin of the experimentally observed transient change of the spectral weight on the Fermi surface? At first glance, few different scenarios could lead to this observation: (i) a simple light-induced population of the originally unoccupied γ pocket concomitant with the finite energy resolution of the experimental setup, (ii) a Floquet-type state from periodically driven lower-lying bands, (iii) a light-induced structural phase transition leading to the modification of the electronic structure, and last, (iv) renormalization of the electronic band structure due to dynamical changes in the effective electronic correlations (transient changes of Hubbard *U*). In the following, we provide clear evidence that only the last scenario is in complete agreement with both our experimental observations and our first-principle simulations.

**Fig. 3:**
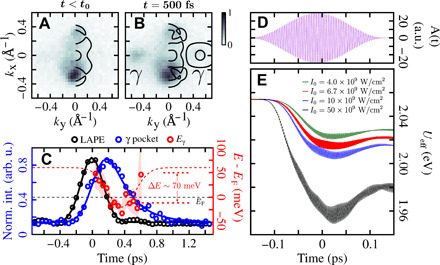
Ultrafast modification of Hubbard *U* at the origin of the dynamical Lifshitz transition. (**A** and **B**) Experimentally measured Fermi surface for *t* < *t*_0_ and *t* = 500 fs and Fermi surface before and after the pump pulse, obtained by TDDFT+*U* simulations using the same intensity as in the experiment (*I*_0_ = 6.7 × 10^9^ W/cm^2^). In both cases (theory and experiment), the γ electron pocket close to the *Y* point, i.e., the hallmark of the Lifshitz transition, is clearly showing up after the interaction with the pump pulse. (**C**) Normalized laser-assisted photoemission signal (LAPE) (black), normalized γ pocket excited state signal (blue), and energy position of the bottom of the γ pocket (red), as a function of pump-probe delay. The shallow red curve represents the 95% confidence intervals of the γ pocket position extracted from EDC fitting. The dashed red curve serves as a guide to the eye. (**D**) The vector potential of the laser pulse used in the self-consistent TDDFT+*U* simulations, with the same wavelength and duration as used in the experiment. (**E**) Dynamical evolution of *U*_eff_ for different laser fluences.

The multidimensional nature of the experimental photoemission data allows us to gain an additional perspective on the laser-induced dynamics by looking at the energy-resolved signal along Y-Γ-Y (see the Supplementary Materials for time-dependent cuts along X-Γ-X). These momentum-energy snapshots reveal the light-induced population dynamics within the γ pockets accompanied by their time-dependent energy downshift. By fitting the time-resolved energy distribution curves (EDCs) at the *Y* point (see Supplementary Materials), we have extracted the position of the bottom of the γ pocket, which is shown to transiently and reversibly downshift in energy by ∼70 meV (Fig. 3C). This dynamical energy downshift of the γ pockets results in a transient crossing of the ground-state Fermi energy by 17 ± 7 meV and causes a disruption of the Fermi surface topology. This effect can neither be explained by a transient excited state population in a rigid band structure picture nor by the finite energy resolution of the experimental setup. This unambiguously rules out scenario (i). Moreover, scenario (ii), involving Floquet-type states, can also be safely ruled out since the measured renormalization of the γ pocket is significantly delayed with respect to the pump pulse (Fig. 3C).

Hence, there remains scenarios (iii) and (iv) as plausible origins of the observed ultrafast Lifshitz transition. As shown in the Supplementary Materials, γ pockets lying slightly below the Fermi energy are also a feature of the *1T*′ phase of MoTe_2_. A photoinduced structural phase transition from the *T*_d_ to the *1T*′ phase would, therefore, lead to the γ pockets crossing the Fermi level upon photoexcitation on the time scale of these structural changes. Zhang *et al.* (*28*) has recently studied this ultrafast lattice symmetry switching (*T*_d_ to *1T*′) using optical techniques by looking at the fluence dependence of coherent phonon modes and of the intensity loss of second-harmonic generation. When pumping above a critical fluence of >2 mJ/cm^2^, they showed that the structural phase transition occurs on the time scale of ∼700 fs and recovers to the *T*_d_ phase within hundreds of picoseconds. The absorbed pump fluence that we used (∼0.6 mJ/cm^2^) is significantly lower than the structural phase transition critical fluence, and we observe a sub–400-fs modification of the Fermi surface topology with a sub–1.5-ps recovery time scale, which does not seem to be compatible with a photoinduced structural phase transition as observed in Zhang *et al.* (*28*). We did not observe any signature of coherent phonons in our experimental data. Moreover, because of the longer wavelength of our pump pulse (1030 nm versus 800 nm), the penetration depth (δ = 410 nm) is more than four times larger than at 800 nm (δ = 100 nm). This means that the pulse energy is deposited on a much larger volume, leading to a much smaller effective temperature increase. Using the equation $S\delta \rho /M\int_{T_{0}}^{T_{0}+\Delta T}C_{p}(T)\text{dT}=FS$, where *S*, ρ, *M*, *C*_p_, and *F* are, respectively, the excitation area, the mass density, the molar mass, the heat capacity, and the absorbed fluence, we can estimate the lattice temperature rise ΔT after the interaction with the pump (*28*). Using the experimental values of ρ, *M*, and *C*_p_ (*29*), we obtain that our laser excitation raises the lattice temperature from *T*_0_ = 30 K to *T*_0_ + ΔT = 71 K, which is well below the critical structural phase transition temperature of ∼250 K. Therefore, the photoinduced structural phase transition [scenario (iii)] can be safely excluded.

The remaining scenario, which fits with all experimental observations, is that the ultrafast Lifshitz transition is of electronic nature and originates from the dynamical modification of *U*. To investigate this scheme in greater details, we have performed first-principle self-consistent TDDFT+*U* calculations (*17*). This method captures both the laser-induced dynamical populations (nonthermal distribution of states) and the time dependence of the effective electronic correlations (dynamical Hubbard *U*). For these calculations, we assume a frozen lattice.

The calculated Fermi surfaces before and after the interaction with the pump pulse, shown in Fig. 3 (A and B), clearly show that the TDDFT+*U* simulations predict the ultrafast dynamical Lifshitz transition, in agreement with the experimental observations. Moreover, as predicted for the strongly correlated charge-transfer insulator NiO (*18*) and a pyrochlore iridate (*19*), the Hubbard *U* is found to decrease upon photoexcitation for *T*_d_-MoTe_2_ (Fig. 3, D and E). This can be understood in terms of dynamical enhancement of the electronic screening due to the delocalized nature of the pump-induced excited electrons (*18*). In the simulations, the modification of the Hubbard *U* seems to reach a plateau after the end of the laser pulse (Fig. 3E), while in the experiment, the energy shift of the pockets is delayed compared to the pump laser pulse, timed by the laser-assisted photoemission signal (Fig. 3C). This may be understood as the effect of other scattering processes, such as electron-phonon coupling, unaccounted for in the simulation that will prevent the laser-driven quenching of *U* from persisting indefinitely. In other words, the simulations intrinsically cannot capture the energy upshift of the pocket at longer pump-probe delays. Moreover, we note that the good agreement (for small pump-probe delays) between theory, which assumes a frozen lattice, and experiment in describing the lowering of the γ pocket is yet another indication that coherent phonons are not playing an important role observed dynamics.

Another interesting observation is that the calculated dynamical reduction of the *U*_eff_ (Fig. 3E) is found to be much smaller than what is expected for reaching the Lifshitz transition from equilibrium calculations (Fig. 1C). To explain why such a small change in *U*_eff_ can induce a Lifshitz transition in the nonequilibrium case, whereas this would not be the case for an adiabatic (equilibrium) scenario, we have to remember that both dynamical populations and time-dependent Hubbard *U* can strongly affect the electronic structure and the Fermi surface of *T*_d_-MoTe_2_. We, therefore, investigated their respective role in driving the nonequilibrium Lifshitz transition (see Supplementary Materials). From this detailed analysis, we found that modifications of both dynamical populations and Hubbard *U* are required to reach the ultrafast dynamical Lifshitz transition. If we freeze the Hubbard *U* in the simulations, then the Lifshitz transition does not occur. We also found that using the adiabatic electronic states to define the Fermi surface after laser excitation does not lead to the Lifshitz transition.

Our TDDFT+*U* framework, which assumes a frozen lattice, cannot quantitatively reproduce the full dynamics from photoexcitation to thermalization, e.g., because of the lack of electron-phonon coupling. However, qualitatively, the fact that this novel nonequilibrium route requires a significantly smaller modification of the Hubbard *U* to reach the Lifshitz transition has strong implications. First, it reveals that nonadiabaticity plays a key role in the observed ultrafast Lifshitz transition. Moreover, our results establish that the synergy between dynamical populations and Hubbard *U* can facilitate reaching topological electronic transitions; in cases where only changing *U*, using adiabatic techniques would not allow it.

In conclusion, using time-resolved multidimensional photoemission spectroscopy, we have demonstrated a fundamentally new nonequilibrium scheme that allows us to drive an ultrafast Lifshitz transition in the Weyl semimetal *T*_d_-MoTe_2_ upon transient and reversible modification of the band structure. Our first-principle simulations revealed the role of the dynamical modulation of the populations and of the electronic correlations in reaching the topological electronic transition on ultrafast time scales. Our work thus demonstrates that dynamical correlations and nonadiabaticity are key ingredients to drive the nonequilibrium Lifshitz transition. This ultrafast topological transition thus finds its roots in different physical mechanisms than more conventional adiabatic Lifshitz transitions. Moreover, the ultrafast Lifshitz transition presented here is of electronic origin, allowing to switch between different Fermi surface topologies and thus to switch the material’s (e.g., transport) properties (*30*) on very fast time scales. Combining this scheme with the emerging field of twistronics (*31*) will allow an unprecedented level of control on the electronic correlations on time scales not accessible to established adiabatic methods.

## MATERIALS AND METHODS

### Time-resolved multidimensional photoemission spectroscopy

The time-resolved multidimensional photoemission spectroscopy experiments were performed at the Fritz Haber Institute of the Max Planck Society. We used a homebuilt optical parametric chirped-pulse amplifier (OPCPA) delivering 15 W (800 nm, 30 fs) at 500-kHz repetition rate. The second harmonic of the OPCPA output (400 nm) is used to drive the HHG by tightly focusing (15-μm FWHM) p-polarized laser pulses onto a thin and dense Argon gas jet. The extremely nonlinear interaction between the laser pulses and the Argon atoms leads to the generation of a comb of odd harmonics of the driving laser, extending up to the 11th order. The copropagating driving laser and the harmonics are reflected onto a silicon wafer at Brewster’s angle of the 400 nm to filter out the energy of the fundamental driving laser. Next, a single harmonic (seventh order, 21.7 eV) is isolated by reflection on a focusing multilayer XUV mirror and propagation through a 400-nm-thick Sn metallic filter. A photon flux of up to 2 × 10^11^ photons/s at the sample position is obtained (110-meV FWHM) (*25*).

As a pump beam, we used a fraction of the compressed 1030-nm pulses used to generate the white light seed in the OPCPA. The combination of longer wavelength and longer pulse duration (compared with 800 nm, 30 fs typically used) allows us to use higher pump fluence before reaching the threshold of pump-induced multiphoton photoemission, which introduces space-charge distortion in the time-resolved multidimensional photoemission measurement. Using high enough fluence is essential to drive the nonequilibrium Lifshitz transition. The drawback is that the temporal resolution decreases to ∼140-fs FWHM. The pump beam was also linearly *p*-polarized (along the Γ-X direction of the crystal) and was at an angle of incidence of 65° to the sample surface normal.

The bulk *T*_d_-MoTe_2_ samples are cooled to 30 K on the 6-axis cryogenic manipulator (SPECS GmbH) and cleaved at a base pressure of 2 × 10^−11^ mbar. The data are acquired using a time-of-flight momentum microscope (METIS1000, SPECS GmbH), allowing to detect each photoelectron as a single event and as a function of pump-probe delay. The resulting 4D photoemission intensity data have the coordinates I(*E*_B_, *k*_x_, *k*_y_, Δ*t*). This represents an improvement with respect to standard time- and angle-resolved photoemission spectroscopy, where 3D photoemission intensity, i.e., I(*E_B_*, *k*_‖_, Δ*t*), is typically measured and where *k*_‖_ is the parallel momentum of the electron in the crystal along one specific direction of the Brillouin zone. The measured *k*_‖_ is determined by the orientation of the crystal with respect to the hemispherical analyzer slit.

Concerning the data after processing, the combination of the high repetition rate of our beamline and the multidimensional data recording scheme (*16*) leads to typical datasets involving 10^9^-10^11^ detected events and a typical dataset size of few hundreds of gigabytes (GBs). We thus use a recently developed open-source workflow (*32*) to efficiently convert these raw single-event–based datasets into binned calibrated data hypervolumes of the desired dimension, including axes calibration and detector artifact corrections. Binning of the single-event data to a specific hypervolume reduces the data to a manageable size of up to a few GBs.

### DFT+*U* and TDDFT+*U* calculations

All the calculations presented in the article and in the Supplementary Materials were performed for bulk *T*_d_-MoTe_2_ unless stated otherwise. Calculations were performed using fully relativistic Hartwigsen-Goedecker-Hutter norm-conserving pseudopotentials using the crystal structure taken from (*33*) (real-space spacing of Δ*r* = 0.158 $\overset{{}^{\circ}}{A}$). Time-dependent calculations have been performed using a 6 × 5 × 3 **k**-point grid to sample the Brillouin zone, whereas ground-state calculations were performed using a 12 × 10 × 6 **k**-point grid. We found that this choice does not lead to a sizable change in the value of the computed Hubbard *U*_eff_. The pump driving field is applied along the (100) crystallographic direction in all the calculations, which corresponds to the Γ-X direction. We considered a laser pulse of 140-fs duration (FWHM) with a sine-square envelope for the vector potential. The carrier wavelength λ is 1030 nm. The time-dependent wavefunctions and Hubbard *U*_eff_ are computed by propagating generalized Kohn-Sham equations within real-time TDDFT+*U*, as provided by the Octopus package (*34*–*36*). We used the local density approximation for describing the local DFT part, and we computed the effective *U*_eff_ = *U* − *J* for Mo *d* orbitals using localized atomic orbitals from the corresponding pseudopotentials (*17*).

We used the real-time TDDFT+*U* formalism (*17*) based on the recently proposed Agapito-Curtarolo-Buongiorno Nardelli (ACBN0) functional (*37*), which can be seen as a pseudo-hybrid reformulation of the DFT+*U* method. The time-dependent generalized Kohn-Sham equation within the adiabatic approximation reads (the nonlocal part of the pseudopotential is omitted for conciseness)

$$\begin{array}{c} i\frac{\partial }{\partial t}|\psi_{n,k}(t)\rangle =[\frac{\hat{p}-A(t)/c}{2}+{\hat{v}}_{\text{ext}}+{\hat{v}}_{H}[n(r,t)]+\\ {\hat{v}}_{\text{xc}}[n(r,t)]+{\hat{V}}_{U}[n(r,t),\{n_{mm\prime }^{\sigma \sigma \prime }\}]]|\psi_{n,k}(t)\rangle \end{array}$$ (1)

where ∣ψ_*n*,**k**_ > is a Pauli spinor representing the Bloch state with a band index *n* at the point **k** in the Brillouin zone, ${\hat{v}}_{\text{ext}}$ is the ionic potential, **A**(*t*) is the external vector potential describing the laser field, ${\hat{v}}_{H}$ is the Hartree potential, ${\hat{v}}_{\text{xc}}$ is the exchange-correlation potential, and ${\hat{V}}_{U}^{\sigma }$ is the (nonlocal) operator

$${\hat{V}}_{U}[n,\{n_{mm^{\prime }}^{\sigma \sigma^{\prime }}\}]=U_{\text{eff}}\sum_{m,m^{\prime }}(\frac{1}{2}\delta_{mm^{\prime }}-n_{mm^{\prime }}){\hat{P}}_{m,m^{\prime }}$$ (2)

Here, ${\hat{P}}_{mm^{\prime }}^{\sigma \sigma^{\prime }}=|\phi_{m}^{\sigma }><\phi_{m^{\prime }}^{\sigma^{\prime }}|$is the projector onto the localized subspace defined by the localized orbitals $\left\{\phi_{m}^{\sigma }\right\}$ and *n*^σσ′^ is the density matrix of the localized subspace, both of these quantities are non-diagonal in spin space (*17*). The expressions of *U* and *J* can be found in (*17*) for the noncollinear spin case.

## SUPPLEMENTARY MATERIALS

Supplementary material for this article is available at http://advances.sciencemag.org/cgi/content/full/7/17/eabd9275/DC1
